# Unusual Metastasis From Breast Cancer: Case Report

**DOI:** 10.7759/cureus.18737

**Published:** 2021-10-13

**Authors:** Laila Jaouani, Adil Zaimi, Ouissam Al Jarroudi, Sami Aziz Brahmi, Said Afqir

**Affiliations:** 1 Medical Oncology, Mohammed VI University Hospital, Oujda, MAR

**Keywords:** transurethral resection, bladder metastases, cystoscopy, hematuria, breast carcinoma

## Abstract

Metastatic breast cancer among young women represents a serious public health issue. The most frequent sites of dissemination are bone, liver, lung, lymph nodes and brain. Bladder location is extremely unusual. We present the case of a 33-year-old female who was receiving palliative chemotherapy for bilateral metastatic invasive lobular cancer. Following episodes of macroscopic hematuria, a CT scan was performed, which revealed a *suspicious* thickening of the bladder wall. After an endoscopic resection with immunohistological analysis, the diagnosis was confirmed. Voiding symptoms in a woman with a history of breast cancer should be evaluated to rule out a secondary urinary tract lesion. As soon as the diagnosis is determined, appropriate therapy should be initiated.

## Introduction

Currently, breast cancer is the most common cancer affecting women worldwide, and its incidence is steadily increasing with 2.3 million women being newly diagnosed each year [1]. While significant progress has been achieved in breast cancer care and treatment results remain better, unfortunately breast cancer remains the second leading cause of cancer mortality among women in all countries, with 685,000 deaths each year [2]. In Morocco, breast cancer is a major public health problem, According to the World Health Organization cancer registry "GLOBOCAN 2020"; 11747 (38.9% of all cancers) new cases were diagnosed [3]. In 2012, the incidence rate in the eastern region of Morocco was 36.8 per 100,000. Lack of screening, late diagnosis, and locally advanced types are nevertheless prevalent, creating management challenges [4]. The most common sites of reported metastases are bone, liver, and lung, as well as lymph nodes and the brain [5]. Only a few cases of bladder metastases have been described in the literature [6]. The most common symptoms are cystitis and macroscopic hematuria. To distinguish primary bladder cancer from a secondary bladder metastasis from breast cancer, a cystoscopy with biopsy is required. We report a new case of bilateral breast cancer with a bladder metastasis occurring during treatment. 

## Case presentation

A 33-year-old female patient with no previous significant history. The initial symptomatology was marked by the onset of dyspnea stage II. A chest X-ray performed showed a right pleural effusion. Pleural tuberculosis was evoked in the first place, considering the endemic character of this pathology in our country. A thoracentesis with cytobacteriological study was performed and revealed no bacterial infection. Investigations were continued by carrying out a staging thoracic-abdominal-pelvic CT scan that showed secondary hepatic, pleural and bone localizations.

The clinical examination revealed a patient with bad performance status, presenting with bilateral and multifocal breast lumps. the left breast lumps localised in the lower inner quadrant and in the upper outer quadrant measuring 35 mm and 30 mm, the right breast characterised by the presence of two lumps in the upper inner and outer quadrant measuring 15 mm and 35 mm with a bilateral axillary lymphadenopathy which was suspicious of malignancy. Mammography and ultrasound revealed breast lesions, classified as category five of the breast imaging-reporting and data system (BI-RADS 5). A biopsy with anatomopathological and immunohistochemical study confirmed the diagnosis of bilateral invasive lobular carcinoma: Scarff-Bloom-Richardson (SBR) grade II, estrogen receptor (ER) at 100%, progesterone receptor (PR) at 20%, human epidermal growth factor receptor 2 (HER2) negative, KI67 at 5%. The initial cancer antigen 15-3 (CA15-3) biomarker was higher with 127 IU/ml.

The patient was treated with anthracycline 60 mg/m^2^, cyclophosphamide 600 mg/m^2^ and zoledronic acid 4 mg every 21 days as a first-line systemic chemotherapy. After six cycles, the CA15-3 decreased to 7.17 IU/ml with favorable evolution of target lesions. The patient began maintenance therapy with Tamoxifen + Medical castration using monthly Goserelin 3.6 mg subcutaneously.

**Figure 1:**
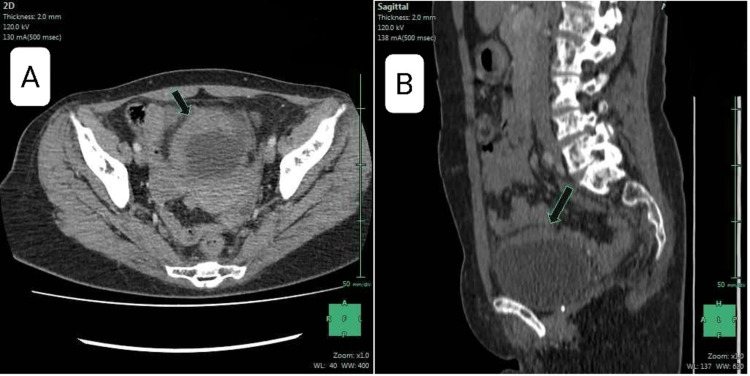
Axial (A) and sagittal (B) contrast-enhanced CT images of the abdomen and pelvis, showing a bladder wall thickening.

**Figure 2:**
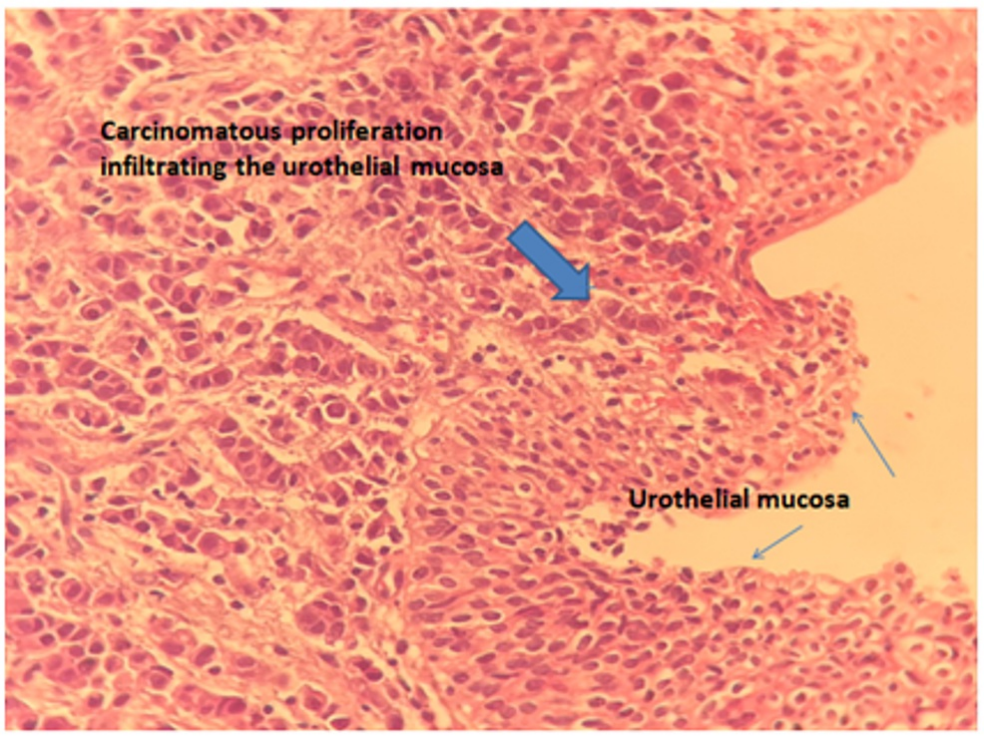
Invasive lobular carcinoma of the breast infiltrating the bladder mucosa.

**Figure 3:**
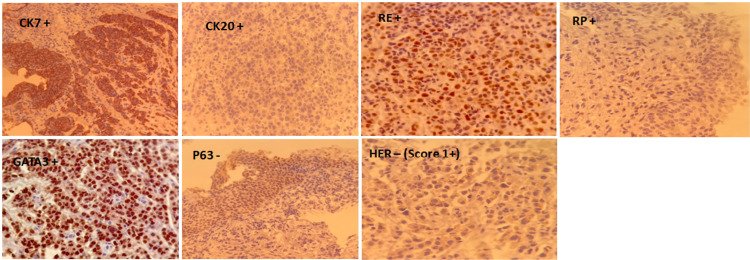
Immunohistochemical findings.

Ten months later, the patient was suffering from macroscopic hematuria, pollakiuria and dysuria. The urine cytology was negative for malignant cells. The CA15-3 at 25 IU/ml. As part of the evaluation, the patient underwent a second thoraco-abdomino-pelvic CT scan which showed stability of the initial lesions and the appearance of a bladder wall thickening (Figure 1). Following an abdominal ultrasound, cystoscopy was performed and a budding mass in the bladder wall was found. The immunohistochemical profile of the biopsies was compatible with a bladder localization of a poorly differentiated carcinomatous process of mammary origin (anti-CK7+, anti-CK20-, anti-P63-, anti-GATA3+, ER+, PR+, HER2) not overexpressed (Figures 2, 3)

The case was discussed in a multidisciplinary consultation meeting. The indication for hemostatic radiotherapy was not retained because the patient was hemodynamically stable without massive bleeding and second-line chemotherapy was started based on docetaxel 75 mg/m^2^ associated with oral tranexamic acid for hemostatic purposes. The patient died of interstitial lung disease 15 days after three cycles of chemotherapy. 

## Discussion

Bladder metastases from solid tumors are very rare and unusual. They represent less than 2% of bladder cancers [7]. In 1950, Abrams et al analyzed, for the first time, the incidence of metastases by autopsy in 1,000 patients. Out of 167 patients with metastatic breast cancer, only four patients had bladder localizations, representing 2.4% versus 77.2% for the lung localization [8]. To date, 65 cases have been published in the literature [6].

Extension to the bladder frequently occurs by contiguity from a pelvic organ such as prostate, cervical and colon cancer. Metastasis from a distant organ is rarely reported in the literature [9,10]. It is thought to be due to hematogenous migration of tumor emboli via the pulmonary circulation [11,12]. Pontes and Oldford propose the hypothesis of bladder involvement with retroperitoneal origin [12].

There are two main histologic subtypes of breast cancer: invasive ductal carcinoma (IDC) accounting for 90% and invasive lobular carcinoma (ILC) for 5%-15% [13]. Although they have a common tropism to bone (50%), brain (16%), lung (17%,) and liver (6%), the lobular subtype has an affinity to the gastrointestinal tract, peritoneum and retroperitoneum [14-16]. This different metastatic potential is explained by the size and shape of ILC tumor cells and the loss of expression of E-cadherin; a molecule responsible for cell cohesion.

Bladder involvement is either synchronous or metachronous with breast cancer. In the majority of cases, the bladder location is part of a multi-metastatic cancer; solitary forms have also been reported [17-19]. The delay in the occurrence of bladder metastasis during the course of the disease is very variable; it can be up to thirty years after the diagnosis of breast cancer [18]. In our case, the patient showed a synchronous bladder metastasis of breast cancer during treatment.

Bladder metastasis poses a problem of differential diagnosis with a primary bladder tumor, clinically, biologically and radiologically. Differentiation between a poorly differentiated or undifferentiated primitive bladder cancer and a bladder metastasis of another primary carcinoma can be difficult [20]. Biopsy with pathological and possibly immunohistochemical study is the key to diagnosis.

The clinical presentation is pleomorphic, ranging from a silent bladder metastasis discovered incidentally during the radiological check-up [20] to a relevant symptomatology. The major warning sign is microscopic and then macroscopic hematuria [21]. Irritative symptoms include pollakiuria, dysuria, polyuria, incontinence and low back pain [18,22]. Renal failure on bilateral hydronephrosis may be a telling complication [23,24]. Urinary disorders depend on the degree of invasion of the bladder wall. Irritative signs appear secondary to detrusor infiltration and may precede hematuria. The appearance of urinary symptoms in a patient with a history of breast cancer should be carefully investigated. Given the risk of delayed diagnosis of bladder metastasis in the case of patients with post-chemotherapy hematuria or cystitis, including cyclophosphamide.

When a patient with breast cancer is experiencing urinary problems, a bladder ultrasound is the first line of investigation. A normal urine cytology does not exclude the diagnosis of a bladder tumor. A CT or MRI scan may also be performed to explore the upper urinary tract in patients with hematuria [25]. PET scan is useful for detecting local recurrence and distant metastases but it has somewhat limited capacity to detect bladder hypermetabolism. In the initial management of breast cancer, the role of CA 15-3 is highly controversial [26]. Its effectiveness in the early diagnosis of breast cancer metastases is recognized. There is a good correlation between the biological and clinical evolution of patients during the treatment of their metastases [27]. 

Cystoscopy is the gold standard examination, performed in the office with a sterile urinalysis. It may find an irregular mass, especially lateral and posterior, a thickened bladder wall and sometimes small polyps [18,28]. Transurethral resection is both a diagnostic and therapeutic procedure. It allows the definitive diagnosis to be established.

In our case, the diagnosis of bladder metastasis was based on the immunohistochemical study which complements the morphological study. The first step of the decision algorithm is based on a battery of markers. The low molecular weight cytokeratins CK7 and CK20 allow a rough orientation towards a primary tumor [29]. CK7 positivity, CK20 negativity and GATA3 positivity suggested the mammary origin of the bladder involvement. GATA3 is expressed in 92% to 100% of ductal and lobular breast cancer. The presence of hormone receptors (ER 20%, PR 10%) and HER2 negativity confirmed the diagnosis of bladder metastasis from a mammary origin. In the literature, studies have shown that a discordance in hormone receptor expression has been reported between primary breast cancer and bladder metastasis [17,18,30,31].

The treatment of metastatic breast cancer has undergone tremendous progress in recent years. Chemotherapy is no longer the only standard treatment for visceral crisis. With the advent of immunotherapy [32], PARP inhibitors [33], CDK4/6 inhibitors and endocrine therapy, progression-free survival and overall survival have been improved [34]. Currently, the combination of CDK4/6 inhibitors (Palbociclib, abemaciclib or ribociclib) and aromatase inhibitor or Fulvestrant [35-37] remains the first-line treatment for hormone receptor-positive metastatic breast cancer (RH+) according to the European Society for Medical Oncology "ESMO" and National Comprehensive Cancer Network " NCCN " guidelines [38].

Our patient received first-line anthracycline-based regimen, which has proven its effectiveness in terms of objective response according to the first meta-analysis by Fossati et al. [39], and zoledronic acid for delaying skeletal events [40]. The duration of chemotherapy in the metastatic setting is until disease progression or excessive toxicity. Maintenance with hormone therapy has shown a good result in terms of progression-free survival and overall survival with fewer side effects [41].

The transurethral resection of the bladder (TURB) has a diagnostic and therapeutic interest while bladder irrigation with tranexamic acid is also effective in emergency situations. Palliative radiotherapy is useful for local control of the disease and especially for haemostatic purposes [42,43].

Unfortunately on the whole, the prognosis of breast cancer with bladder metastasis is poor. Overall survival varies between one month and two years [44]. This is the case of our patient who died four months after diagnosis.

## Conclusions

Although secondary bladder tumors are uncommon, they should be suspected in any patient with a history of breast cancer who presented urinary disorders. The most common symptom of the illness is hematuria. Cystoscopy with biopsy and immunohistochemistry is the critical stage in the diagnostic procedure for distinguishing between primary and secondary malignancy.
